# Watchfully checking rapport with the Primary Child Health Care nurses - a theoretical model from the perspective of parents of foreign origin

**DOI:** 10.1186/1472-6955-9-14

**Published:** 2010-07-20

**Authors:** Anita Berlin, Lena Törnkvist, Ingrid Hylander

**Affiliations:** 1 Centre for Family and Community Medicine, Department of Neurobiology, Care Sciences and Society, Karolinska Institute, Alfred Nobels Allé 12, SE-141 83 Huddinge, Stockholm, Sweden

## Abstract

**Background:**

Worldwide, multicultural interaction within health care seems to be challenging and problematic. This is also true among Primary Child Health Care nurses (PCHC nurses) in the Swedish Primary Child Health Care services (PCHC services). Therefore, there was a need to investigate the parents' perspective in-depth.

**Aim:**

The aim of the study was to construct a theoretical model that could promote further understanding of the variety of experiences of parents of foreign origin regarding their interaction with the PCHC nurses at PCHC services.

**Method:**

The study used Grounded Theory Methodology. Twenty-one parents of foreign origin in contact with PCHC servicies were interviewed.

**Results:**

In our study parents were watchfully checking rapport, i.e. if they could perceive sympathy and understanding from the PCHC nurses. This was done by checking the nurse's demeanour and signs of judgement. From these interviews we created a theoretical model illustrating the interactive process between parents and PCHC nurses.

**Conclusion:**

We found it to be of utmost importance for parents to be certain that it was possible to establish rapport with the PCHC nurse. If not, disruptions in the child's attendance at PCHC services could result. PCHC nurses can use the theoretical model resulting from this study as a basis for understanding parents, avoiding a demeanour and judgements that may cause misunderstandings thus promoting high-quality interaction in PCHC services.

## Background

Multicultural interaction in health care between clients and caregivers has been described as challenging and problematic worldwide [1]. These challenges were also found in the Swedish Primary Child Health Care services (PCHC services) [2-4]. Sweden is a multicultural society. In the capital, Stockholm, more than 20% of the population was born in another country [5] and one third of children seen by the PCHC services in Stockholm County are of foreign origin [6].

A recent study found that a majority of Primary Child Health Care nurses (PCHC nurses) experienced difficulties in their interactions with children and parents of foreign origin [2]. An in-depth analysis of these experiences revealed that PCHC nurses have difficulties performing one of their main tasks - assessing health risks in the children's psychosocial home environment. PCHC nurses were unfamiliar with and had difficulty understanding the families' living conditions and how parents approached their children. Thus, they became uncertain about making decisions, and so continued to assess health risks while conducting different strategies [3]. Consequently, this process could be time-consuming and delay identification of health risks to children of foreign origin and of interventions to secure optimal development of physical, mental and psychosocial health. It might also risk disturbing the relationship between the PCHC nurses and the families [3]. The conditions described above are alarming in two respects. First, early identification of psychosocial ill-health in children is stressed as a task of general importance in the Swedish PCHC services [7] and must be seen as particularly important when dealing with immigrant families. It is well known that many of these families are exposed to heavy psychosocial stress related to experiences in their home countries (war and persecution), migration [8,9] and segregation in the new country [10,11]. These circumstances are known to negatively impact the health and well-being of the whole family [11]. Second, to promote health and detect and respond to special health care needs in these children, the PCHC services must be skilful in their interaction with parents from diverse cultures. Provision of high-quality PCHC services on equal terms requires new knowledge of parents' experiences with this interaction, particularly their thoughts about how optimal cooperation with the PCHC nurse could be described. Previous studies on ethnic diversity in relation to child health care services have mostly investigated broad issues such as access to services [8,12] and risk factors for, and barriers to, satisfaction [11,13]. Because earlier studies [2,3] revealed that PCHC nurses experience difficulties when interacting with children and parents of foreign origin, the parents' perspective now needs to be investigated.

The aim of the study was to construct a theoretical model that could promote further understanding of the variety of experiences of parents of foreign origin regarding their interaction with the PCHC nurses at PCHC services.

## Methods

### Definition

The concept 'parents of foreign origin' refers to parents born outside the Nordic countries (i.e., Sweden, Norway, Denmark, Finland and Iceland), a definition taken by the PCHC services in Stockholm County [6]. For the sake of brevity, these parents of foreign origin will be referred to simply as 'parents' in this paper.

### Setting

PCHC services have a long tradition in Sweden. They are available on a voluntary basis at no cost, with a participation rate approaching 100% [7]. These services deal with most aspects of children's health, and provide health checks for children aged 0-6 years and their parents, according to a general core program [14]. PCHC services are provided at Primary Child Health Centres (PCHC) by PCHC nurses, who are the key people for most child health activities, such as health education, vaccinations, psychosocial support and advice. They perform health examinations including assessing the child's motor and linguistic development as well as his/her physical, mental and psychosocial health (including the family's social situation). Assessments are done to determine the type of intervention or individual support a child and family may need for the child's optimal physical, mental and psychosocial health development. PCHC nurses are obligated to alert the social services if they believe that a child may need protection [7,15].

### Design

The study used the Grounded Theory Methodology (GTM), because it focuses on interactions and social events [16-18] and aims to generate theories and concepts grounded in empirical data [19] as well as dealing with interactions in their natural and daily context. In this study, that means parents and their interactions with PCHC nurses in PCHC services. The type of grounded theory method used is close to classic grounded theory in keeping close to data and emphasising conceptualisation [18]. When it comes to ontology, however, we rely on a constructivist approach [19] and recognise the roots from symbolic interactionism [16]. This approach is outlined in Hylander [17]. For recent examples see Berlin et al. [3] and Modin et al. [20]. Accordingly, the outcome of the study - the theoretical model - is a construction by the researchers, which is only a set of hypotheses, although well grounded in the data.

### Data collection and participants

From June 2006 through March 2007 individual face-to-face interviews were done by the lead author. The lead author spent time in the waiting rooms of six different PCHCs on seven occasions, contacted parents and presented written and oral information about the aim of the study. Parents who were not dependent on an interpreter were selected. The parents decided whether they wanted to be interviewed and where. The interview guide was semi-structured with open-ended questions (Table 1). Interviews ranged in length from 30 to 65 minutes, were tape-recorded and transcribed verbatim. Memos were written right after each interview.

**Table 1:** Open-ended interview questions

	Initially, the parents were asked to describe their experiences when interacting with the PCHC nurses within the following themes:
1.	Scenarios (episodes/incidents) of interactions

2.	Actions taken/feelings/thoughts in different situations, and how the parents handled/felt/thought in different episodes/incidents.

3.	What the parents considered to be important factors in the interaction.

Theoretical sampling was conducted to acquire variation in data and fill the categories of the emerging model. First, two first time parents were selected with children under the age of 6 months who frequently visit the PCHC. Second, the same interview guide was given to two more parents with the same characteristics. Third, the interview guide was changed and expanded to cover newly discovered issues and was given to seven parents with more and older children. This selection was done to gain information from parents with more varied experiences with PCHC. Fourth, one parent using an interpreter was chosen. Finally the last sample of 10 parents with different countries of birth were chosen to give their view on fit, work and relevance of the theoretical model.

Twenty-one parents contributed to the comprehensive theoretical model resulting from this study. These parents had a variety of characteristics, i.e., countries of birth Africa (6), Middle East (11) and South Europe (4), sex (17 mothers, 4 fathers), time in Sweden (range 1 - 22 years), number of children per parents (range 1-5) and age of the children (range 3 weeks - 13 years).

### Analysis

A process of constant comparison was carried out throughout the collection, selection, coding and analysis of data until saturation was judged to be reached and concepts had become satisfactorily refined. Memos were written as a basis for decisions concerning areas to cover in further interviews, including specific questions [17]. In the *open coding*, transcripts were read line by line and incidences coded. The parents' own words were used as much as possible in order to capture the *substance of data*. Comparisons were made incident by incident, similarities and differences were identified, and new codes were condensed into categories, which in turn were developed into concepts such as *open, unclear *and *closed demeanour*. In *theoretical coding*, patterns and connections emerged from empirical data in response to the question 'how do the concepts relate to each other?'[17,18]. In *selective coding*, data were coded against the basic social process of parents *watchfully checking for rapport - *i.e., sympathy and understanding. A theoretical model was developed and ten new parents were interviewed. They were asked four specific questions (Table 2) covering the criteria of *fit, work *and *relevance *[21]. To test the saturation of the categories they were asked for suggestions of experiences.

**Table 2:** Results (in sum) from interviewing 10 parents regarding the theoretical model

Questions	Yes	No
	(n)	(n)
1. Do you recognise the experiences illustrated in this model?	10	0

2. Is anything new?	10	0

3. Is the model clear and easy to understand?	10	0

4. Do you have use for the model?	10	0

### Ethical approval

The Ethical Committee of Huddinge University Hospital, Karolinska Institute approved the use of human subjects in this study (registration number 419/03). At the visit, parents were given written and oral information concerning aim of the study, explaining that participation was voluntary, that data would be treated confidentially and that individuals could not be identified from the results. For that reason identifications of the quotations have been omitted. Parents who gave verbal informed consent were invited to participate. The interviewer, i.e., the first author, was the only person who knew the participants' forenames. Tapes were destroyed shortly after the transcriptions and following the guidelines are kept in safety lockers ten years by a secretary with no connection to the project

## Results

The theoretical model resulting from this study illustrates the interaction between the parents and the PCHC nurses and explains the variation in parents' perception of the child's health check-ups. Parents come to the PCHC with a general *feeling of exposure *and *anxiety about being misjudged *as parents due to their origin. Thus, they watchfully check the possibility of establishing rapport with the PCHC nurse by checking the nurse's demeanour and signs of whether and how they are being judged as parents.

### Watchfully checking for rapport

Feelings of exposure and anxiety about being misjudged created caution in the parents and affected how they perceived the PCHC nurse's demeanour and how the interaction with the PCHC nurse proceeded.

Changes in the parents' social situation and feelings of loneliness and anxiety about coping without their network, along with linguistic barriers, made things worse since they could not express themselves or understand what was going on around them. Most things felt strange, different and contrary. The parents hoped that the PCHC nurse would be knowledgeable and aware of their vulnerability.

I wonder if the nurses have thought about how we are so alone that we might need a bit more consideration.

PCHC nurses could learn that not everything that is topsy-turvy is our fault; lots of people have come from war-torn countries with a lot of other terrible stuff that they were exposed to.

They were also concerned that the PCHC nurse was negatively 'influenced' by having heard negative things about, and met, loads of 'foreigners' who had done a lot 'wrong'. It was possible to distinguish factors where, according to parents, there was a risk that they would be misjudged by the PCHC nurse and regarded as a 'stupid and ignorant foreigner'. For example: ask too many and 'wrong' questions, do 'wrong' things with the child, miss appointments or visit the PCHC without an appointment. They felt that they knew a lot about looking after children but were unsure what the PCHC nurse thought of their ways. They felt that views collided and that it was unclear who was right and who was wrong, for example about folk medicines from their home country.

Perhaps the PCHC nurse thinks, 'ah, aniseed, that might not be good for the child'.

### Checking demeanour

Checking demeanour was a quite rapid process in which the parents were watchful of the nurse's facial expressions, gestures and movements and as a result felt secure, confused or vulnerable.

#### Open demeanour - parents feel secure

General impression of open demeanour: A smiling and relaxed facial expression was interpreted as an *open demeanour *and was the primary demeanour described by the parents. According to the parents the nurses had an 'open and warm heart', greeted them warmly, were relaxed, gentle, calm, cheerful, angelic, considerate, accessible, 'easy to reach' and seemed interested in their children. The PCHC nurse joked, laughed and took time to talk. Parents felt secure, calm, bold and unafraid in their contact with the nurse.

 She is always smiling and always says: Hello, how are you?

My PCHC nurse is great, she is nice and funny. I have never felt sad with her....

I like coming here to talk to her.

#### Unclear demeanour - parents feel confused

General impression of unclear demeanour: Parents said that they felt and saw that the PCHC nurse was uncertain and hesitant. They said that she moved anxiously about the room, looked for answers in a book or record, or went and asked someone else.

We see that they are unsure of themselves; we feel that they are unsure.

She doesn't answer properly, starts looking in the book, dashes back and forth...she has to read the child's medical record... 'wait, I'll just check'... or goes and asks someone else.

Parents said that they felt confused by this lack of clarity and had only a vague idea of the job of the PCHC nurse. When parents felt that they did not get what they needed, for example, help in arranging a prescription or a doctor's appointment when the child was ill, they sometimes saw the PCHC nurse as unwilling and lacking knowledge.

She (the PCHC nurse) doesn't know anything because she hasn't prescribed medicine, not even cough medicine.

#### Closed demeanour - parents feel vulnerable

General impression of a closed demeanour: Patients felt that the PCHC nurse was hard to make contact with, did not talk much, was not cheerful or considerate, 'gave negative looks', was in a hurry, was stressed, angry, irritated, task-focused, and described as 'doing her job, no more'.

When she was finished, all that remained was for us to dress the baby and get out of there.

Examinations of the child were done without respect and without first informing the parents.

She never asked me anything, just does anything with my child. That scares you.

Parents felt vulnerable and anxious when they felt that the PCHC nurse did not seem to 'care very much', when they felt that she was very busy and when they did not dare ask questions in case they were labelled stupid and ignorant.

When I ask the PCHC nurse, it's not because I am stupid and ignorant, it's because I want a little more information and explanations so I can make things better for my children.

### Checking signs of judgement

Parents checked for signs of how they were being judged as parents. They were watchful of how the PCHC nurse approached them and asked questions and as a result they felt accepted, insecure or questioned.

#### Giving feedback - parents feel accepted

The parents felt that the PCHC nurse asked for their views and feelings, provided *feedback *through praise, was 'straightforward' and explained her viewpoint clearly.

When the parents felt welcome, remembered and confirmed, the PCHC nurse was perceived to like co-operating with, and be competent in working with foreigners.

I've always felt welcome here; I've never met anyone who was irritated or racist. Maybe she has learned what it's like to work with foreigners?

The parents felt accepted when they were confirmed. They learned new things, grew and felt 'knowledgeable'.

You feel so good when they say you're a good parent.

#### Exploring - parents feel insecure

The parents felt that the PCHC nurse asked pointless questions and gave advice with no explanation. A majority of parents did not wish to, or know that they could, discuss psychosocial problems at the PCHC. That type of problem was seen as too personal. When the nurse asked questions about the family's social life, parents wondered, 'why is she saying that? What do they want? What is she doing? What are they checking? I don't understand.' Parents asked themselves:

They can't just ask, they have to tell us more about why they are asking questions like this.

When asked exploring questions, the parents saw the PCHC nurse as lacking in knowledge and needing to take a course and learn how to work with 'foreigners' because she asked unclear questions and explained poorly.

Maybe she needs to learn what it's like to work with foreigners?

The parents felt insecure and anxious when they sensed the PCHC nurse's uncertainty. They felt that they needed to know what the PCHC nurse really thought, and they did not understand what they could expect of her.

We worry when they work like that, we get insecure too, perhaps it's right but we get insecure.

#### Fault-finding - parents feel questioned

Fault-finding was described as acting like a teacher, transmitting a 'right or wrong' message, perfectionist, exaggerating tiny things and wanting to be in charge. The advice provided was not felt to be adapted to the parents' previous experiences.

When you're there and ask something, maybe you get their look or answer... their answers give us an idea that no, this... you are not to do this, you must do this.

The parents asked themselves whether the nurse's fault-finding could be because the PCHC nurse had been 'affected' by having met many immigrants who did things 'wrong', had gained a negative view and therefore disliked working with foreigners.

I thought, she has seen a lot of foreigners. She doesn't have any more energy to stand there talking.

She doesn't like working with foreigners.

The parents *felt questioned *and 'excluded' when they felt that they were being corrected: they were ashamed and did not understand what they had done 'wrong'. When the PCHC nurse became too detailed in her advice, they thought: 'Are you going to teach me? I already know this!'

Some things feel like, yes but I already know this, and when they sit and tell you, it makes you feel that they think we don't know anything and we know quite a lot...Unfortunately, this is what they think!

### Parents' experiences with the child's health check-up

Parents' watchfulness due to feelings of exposure and anxiety about being misjudged as parents changed depended on the outcome of their checking demeanour and checking for signs of judgement. How parents perceived the child's health check-ups was affected by rapport, which was understood as the experience of mutual sympathy and understanding. Rapport may be possible, uncertain or impossible to establish, and as a result they felt convinced, hesitant or unwilling to go to the child's health check-up.

#### Rapport possible - feeling convinced

Parents' feelings of exposure and anxiety about being misjudged disappeared when they felt secure with, and accepted by, the PCHC nurse. They didn't need their watchfulness but experienced a mutual dialogue, consideration and understanding. It was thus *possible to establish rapport *with the PCHC nurse.

It feels as if we understand each other, parents and PCHC nurse working together.

She (the PCHC nurse) asks questions and seems to understand... it's a two-way thing between us.

Parents felt convinced that the health check-ups at the PCHC were natural and useful because they learned how the child was feeling and developing. The advice given by the PCHC nurse was helpful and viewed as trustworthy. When they compared PCHC services to the health services in their home countries, they thought that the PCHC services were correct.

This is good for the whole of Sweden, for me and my child.

There's nothing strange about it...you come here to check your child and you find out.

#### Rapport uncertain - feeling hesitant

Parents' feelings of exposure and anxiety about being misjudged remained when they felt confused and insecure in relation to the PCHC nurse.

It gets complicated for us if nobody really tells us.

I should ask her, how far can I go with my questions to you? Should I do that?

Here, then, they were *uncertain *about whether they could *establish rapport *with the PCHC nurse.

You don't really know what she's thinking and feeling.

Parents felt *hesitant *about the usefulness and point of the child's health check-ups and what they were given at the PCHC.

The insecurity feels hard for us, the children are really important to us.

This *hesitancy *created anxiety about the child's health that led parents to ask an older relative or neighbour, or find another caregiver.

It's this problem, we are looking for other doctors and nurses or so, at the same time.

##### Rapport impossible - feeling unwilling

Parents' feelings of exposure and anxiety about being misjudged were made worse when they felt vulnerable and questioned in relation to the PCHC nurse. It was thus *impossible to establish rapport *with the PCHC nurse.

I thought, she doesn't understand or care very much about what it's like for us.

The parents perceived the child's health check-ups as unpleasant. They became *unwilling *to go to the PCHC.

If I meet someone and feel that they are closed, like this - (holds up hand in a 'stop' gesture) - I don't want to come back to the PCHC tomorrow.

If the PCHC nurse is negative, you feel unwilling instead of wanting to learn, you feel much more ashamed and feel, OK, well, if we go back it'll be the same... that's what they (PCHC nurses) think about us.

**Figure 1:**
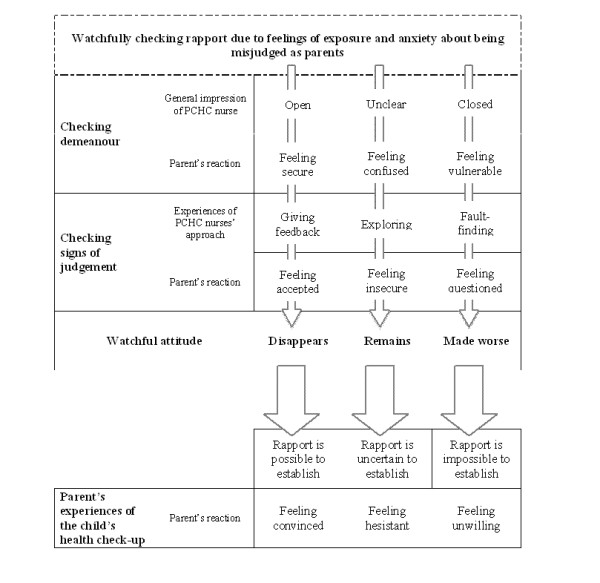
**Parents of foreign origin watchfully checking rapport with the Primary Child Health Care nurses (PCHC nurses)**.

Hence, on the basis of their watchful checking of rapport, the parents concluded that rapport was *possible to establish *and thereby the watchfulness *disappeared *or was *uncertain *and the watchfulness *remained *or it was *impossible *and the watchfulness was *made worse*. Parents concluding that rapport is uncertain or impossible are *hesitant *or *unwilling *to attend the PCHC check-ups. This interactive process between PCHC nurses and parents is illustrated in figure 1.

## Discussion

This study investigated parents' views of interacting with PCHC nurses in PCHC services and revealed the social process of parents *watchfully checking rapport *with the PCHC nurse. The theoretical model illustrates an interactive process in which parents are checking the PCHC nurses' demeanour and checking for signs of being judged in order to find out whether it is possible to establish rapport. The main finding is the importance of parents perceiving rapport, i.e., sympathy and understanding being possible to establish with the PCHC nurse. Hence, it benefits the child's attendence in the PCHC services when parents feel convinced that the child's health check-ups at PCHC are useful. In contrast, if parents perceive rapport to be uncertain or impossible to establish, parents feel hesitant and/or unwilling to take the child to health check-ups.

In literature the noun *rapport *is defined as 'a feeling of sympathy and understanding, emotional bond' [22]. It is a concept used in patient-caregiver relations and psychotherapy [23,24]. Rapport is described as 'key' in building or developing trust and in a positive perception of the interaction [25,26]. Norfolk et al. stressed empathy as essential when establishing rapport [27]. It involves verbal and nonverbal behaviour and Tickle-Degnen & Rosenthal delineated the nonverbal components of rapport as being consistent across a variety of contexts: these are mutual attentiveness, positivity and coordination between participants [23]. Literature stressed the importance of establishing rapport, i.e., developing an alliance with the client to achieve effective treatment [28] and to improve health care, e.g., treatment for substance abuse [29], doctor and client consultation [27], home care for the elderly [30] and children [31].

When parents in our study were watchfully checking rapport with the PCHC nurse it seemed to be a quite rapid process caused by feelings of exposure and anxiety for being misjudged as parents. They were extra observant of the PCHC nurses' facial expressions and body language and got a general impression of PCHC nurses being either open, unclear or closed. When this checking resulted in a belief that rapport could be attained, this might have features in common with 'moments of meeting' described by Stern et al. as an unconventional way of affective 'tuning in', a mutual recognition, mutual understanding transmitted by a glance, word or gesture, leading to a shared implicit relationship[32].

Negative consequences caused by migration and language barriers suggest that it is very important to get sympathy and understanding from a health care provider. From literature it is well known that levels of stress are linked to migration. Heavy personal and socioeconomic losses [9], exclusion due to language barriers, unemployment and bad housing [10], and acculturation stress if accommodation to a dominant culture is forced are described [33]. This seems to be consistent with what parents in our study expressed during the interviews. In addition, parents were anxious about being misjudged because of their foreign origin. Parents said they could understand that nurses might have difficulties with other parents who did not know the language and with whom it was difficult to communicate. They did not want PCHC nurses to generalise and categorisethem as one of 'those immigrants', and emphasised differences between immigrants in education, language skills, etc. Nor did they wish to be held responsible for 'wrong things' other immigrants did with their children. The conclusion of this is a call for education in cultural competence for health care providers to assist in developing a trusting client-nurse relationship, which is in line with well-known recommendations [34-36].

A PCHC nurse's open demeanour was related to parents' positive perception of the interaction. Parents felt comfortable with the PCHC nurses, seemed to understand the nurses' professional role, considered them knowledgeableand interested in them as foreigners. This open demeanour, resulting in the establishment of rapport, is close to discussions in the literature regarding empathy [27]. Ethnocultural empathy is considered a specific type of empathy, particularly in reference to different professional groups in their encounters with patients and clients of different cultural and ethnic origins [37]. One could perhaps conclude that the PCHC nurses perceived by parents in our study as open and warm, willing to understand, giving feedback and communicating openly and straightforwardly, scored high in this type of empathy.

Differences in the cultural backgrounds of parents and PCHC nurses, e.g., implicit expectations and customs of how to respond to a healthcare provider, might explain feelings of insecurity and parents feeling questioned. In a study among Latinos, Flores et al. found that healthcare providers are expected to have a positive attitude. A relatively neutral attitude (possibly the same as unclear or closed demeanour) could be interpreted negatively [12]. Moreover, one way for Mexicans to show respect to a healthcare provider is to not ask questions, since to do so is to question authority [38]. This might lead to feelings of insecurity if the healthcare context of the majority of society is unclear to the client. Parents who felt that PCHC nurses had an *unclear demeanour *might misunderstand the PCHC nurse's professional role, expecting to meet a medical expert who can prescribe medicine and not someone exploring their family situation.

PCHC nurses' psychosocial assessments of health risks for children is a task that may cause tension and ethical dilemmas [4] and is experienced as difficult to perform for children with parents of foreign origin. When unsure they used different strategies to clarify the situation [3]. However, some of these strategies may be experienced as fault-finding by the parents if not done in an open, warm and straightforward way and hence instigate a vicious circle of mutual hesitance and insecurity in the interaction between the parents and the PCHC nurse.

PCHC nurses perceived as unclear and/or closed caused disruptions in the interaction due to parents' being uncertain whether it was impossible to establish rapport. These parents became hesitant and/or unwilling to attend the child's health check-ups at the PCHC. This might have negative consequences: first, risk of health care disparities in a child that needs support and preventive health care from the PCHC services. Second, a risk for misuse of health care resources. Hence, it is known that parents who are poorly treated by health care providers are nearly twice as likely to have used emergency departments when seeking medical advice for their children [39].

### Credibility and limitations

Rigour was obtained by transcribing all interviews immediatly after the interview in addition to memos and by constantly comparing new categorires with raw data and new data with established categories, in the progressing analyses. The last ten parents giving interviews on 'fit, work and relevance' [21] confirmed the social process to be relevant, and the model to be a new and useful way of describing their experiences. No new categories were derived from their reports. Therefore, the theoretical model could be regarded as consistent and clinically relevant in this specific setting. Saturation was judged to be reached, with certain limitations. The categories of unclear demeanour, closed demeanour, exploring and fault-finding could have been further explored by sampling parents who did not frequently attend the PCHC.

The first author has professional experience from the field, which is an advantage in terms of having knowledge of the health care context. On the other hand, preconception might constitute a bias. Therefore memos were written, collaborative analyses with researchers from other disciplines were undertaken and results were debated at several academic seminars.

Interviewing has its limitations and weaknesses especially when respondents are speaking a second language [40]. To diminish these limitations, parents fluent in the Swedish language were prioritised. This strategic sampling may have had a significant impact on the results, which certainly is a limitation in need of more clarification and new research. In one interview an interpreter was used, giving data that did not essentially contribute with new or extended information to the model. Indeed, getting information from non-Swedish speaking parents would require thougtful consideration in the study design.

Moreover, the theoretical model of this study is a substantive theory applicable to the area from which it emerged, i.e., the experience of parents in relation to PCHC in a suburb of a large city in Sweden. It should be regarded, not as a tested hypothesis, but as a set of proposals that are very well grounded in and supported by empirical data. It is likely, however, that the model can be tested in similar settings and modified on detection of new variations.

## Conclusions

Our results indicate that the general impression parents get from the PCHC nurses' demeanour is critical for further interaction and for the child's attendance at the PCHC services. If something goes wrong, it might undermine the most ambitious health care plan, with negative consequences for the child's health. Therefore, PCHC nurses need to be aware of the importance of having an open, sympathic and understanding approach.

This is especially important for parents with feelings of exposure and anxiety to feel secure and accepted. To emphasise high-quality interaction in PCHC services, the concept *open demeanour *could be used as a quality indicator for clinical practice.

The theoretical model could be used as a tool when supervising on this topic to raise the PCHC nurses' awareness of their facial expressions and demeanour and stimulate them to consider their attitudes and prejudices. The model might also serve as a basis for reflection on intercultural issues in training public health and paediatric nurses.

## List of abbreviations used

PCHC services: Primary Child Health Care services; PCHC nurses: Primary Child Health Care nurses; PCHC: Primary Child Health Centres

## Competing interests

The authors declare that they have no competing interests.

## Authors' contributions

AB and LT acquired funding. AB, LT and IH designed the study and constructed the interview protocol. AB collected the data. AB, LT and IH analysed and categorised data. AB drafted the manuscript. LT and IH have critically revised the manuscript. All authors have read and approved the final manuscript.

## Pre-publication history

The pre-publication history for this paper can be accessed here:

http://www.biomedcentral.com/1472-6955/9/14/prepub
